# A conceptual model for building program sustainability in public health settings: Learning from the implementation of the program sustainability action planning model and training curricula

**DOI:** 10.3389/frhs.2023.1026484

**Published:** 2023-03-29

**Authors:** Sarah Moreland-Russell, Eliot Jost, Jessica Gannon

**Affiliations:** Prevention Research Center, Brown School, Washington University in St. Louis, St. Louis, MO, United States

**Keywords:** program sustainability, implementation science, conceptual model, tobacco control programs, agile science

## Abstract

**Background:**

The emergence of implementation science has driven an increase in research examining the implementation of evidence-based programs and policies. However, there has been less attention through program sustainability. To achieve the full benefit of investment in program development and implementation, there must be an understanding of the factors that relate to sustainability; additionally, there is a need for a robust set of tools and trainings to support strategic long-term program sustainability. This paper presents results of our sustainability training intervention and a new conceptual model of sustainability. The proposed conceptual model builds upon the intervention design, further specifying the implementation strategy, strategy-mechanism linkages, and effect modifiers.

**Methods:**

This research is part of the larger randomized control trial evaluating the effectiveness of the Program Sustainability Action Planning Model and Training Curricula. Specifically, this multimethod study establishes a conceptual model for program sustainability and related capacity-building interventions. The training intervention was delivered through workshops and technical assistance to 11 state tobacco control programs, principally entailing the development and implementation of a sustainability action plan. We utilize descriptive statistics and participant perspectives to evaluate the training intervention and propose an empirically-grounded conceptual model for sustainability capacity-building interventions in public health settings.

**Results:**

Participants found intervention components (workshop, workbook, instructor and resources) to be effective. Overall, participants found the intervention improved their ability to develop sustainability action plans and assess their program and partners. Throughout the study, program managers emphasized the importance of the workshop in providing direction for their sustainability work and the value of robust, ongoing technical assistance. Program managers identified several factors that interfered with intervention reception including staff turnover, competing priorities, partnership challenges, and the COVID-19 pandemic.

**Conclusion:**

The present study documents the development and implementation of a novel Program Sustainability Action Planning Model and Training Curricula, one of the first interventions designed to improve program sustainability. In addition, we present an empirically-grounded conceptual model for program sustainability. Considering the paucity of research in this understudied and undefined topic area, this is an important contribution that can serve as a framework for similar intervention designs and implementation efforts.

**Clinical Trail Registration:** ClinicalTrails.gov identification number is NCT03598114.

## Introduction

The emergence of dissemination and implementation (D&I) science has driven an increase in theoretical and empirical understanding of evidence-based program and policy implementation. However, D&I science has paid less attention through the post-implementation period of sustainability. To achieve the full and continued benefits of significant investment in public health research and program development, there must be an understanding of the factors that relate to program sustainability in the post-implementation period; additionally, there is a need for a robust set of tools and trainings to support strategic long-term program sustainability (1).

Despite a burgeoning implementation science literature, there is still a lack of planning for sustainability of evidence-based programs. In a recent review of dissemination and implementation research studies funded by the National Institutes of Health, Johnson et al., found that only only 67.1% of the studies made references to sustainability and none referred to sustainability planning (2). Similarly, there remains a lack of a formally agreed upon definition or elements of sustainability. In Johnson et al.'s review, researchers who actually referenced sustainability in their study, conceptualized sustainability as the “continued delivery of interventions, programs, or implementation strategies,” (2) but there was no formal consensus on the definition. Shediac-Rizkallah and Bone conceptualize sustainability broadly as “*the maintenance of health benefits over time*” (3). Scheirer and Dearing focus exclusively on the organizational and programmatic elements: “*the continued use of program components and activities for the continued achievement of desirable program and population outcomes*” (1). In an attempt to define a more formal definition, Moore et al. (4) abstracted sustainability definitions from 209 articles and mapped constructs from the definitions to create a revised definition of sustainability: “*after a defined period of time, a program, clinical intervention, and/or implementation strategies continue to be delivered and/or individual behavior change (i.e., clinician, patient) is maintained; the program and individual behavior change may evolve or adapt while continuing to produce benefits for individuals/systems”* (4).

We have adopted a more comprehensive definition that considers program organizational components, the evidence for program effectiveness, as well as the process or system in which a program is implemented over time. Comprehensively defined, sustainability is the existence of structures and processes within an adaptive system that allow a program to effectively implement and maintain evidence-based policies and activities that improve health over time (5). This definition is deliberately broad, and moves beyond the characteristics of the program itself to include organizational and other system characteristics. This sustainability definition contains three key elements. First, sustainability is an ongoing (cyclical) change process that requires action-oriented planning to strengthen system capacity (6, 7). Systems include the program, the auspice organization, the community, and the funder. Second, programs rely on structures and processes that contribute toward adequate system capacity as a necessary condition for program sustainability (8). A sustainable program must be integrated into normal organizational operations (9). The characteristics of these programmatic and organizational structures, processes, and community and funder supports (10) build programmatic capacity for sustainability and institutionalization, over time. Finally, what is to be sustained is an evidence-based innovation which is part of a prevention system. Because the innovation is evidence-based, sustainability is essential in attaining positive health impacts (11).

In addition to consensus on one formal definition of sustainability, there remains a lack on congruence in defining “what” factors contribute toward sustainability and “how” programs can achieve sustainability. In considering the factors that contribute toward sustainability Luke et al., identifies 17 frameworks suggesting a variety of factors (with some similarity) that influence program sustainability (12). In addition, only a few conceptual models focus exclusively on the “how” or the programmatic process for building capacity for sustainability. While these frameworks exist, few are actually utilized in D&I research; few researchers funded by the National Institutes of Health referenced frameworks with sustainability constructs and offered limited information on how they operationalized frameworks (2). The Dynamic Sustainability Framework offered by Chambers et al., considers the context in which an intervention is implemented and operationalized within a system (13). However, it does not offer an implementation strategy or mechanism for which programs should engage to improve sustainability of the intervention. May et al.'s, Normalization Process Theory explains how new ideas, ways of acting, and ways of working become routinely embedded or normalized in practice settings (14). It has been utilized in studying program implementation and sustainability (15) and found useful in identifying processes that are likely to enhance sustainability, but again does not offer a mechanism for which programs should engage to improve sustainability. The Program Sustainability Framework (5), which was utilized for our study, outlines eight domains of sustainability including organizational capacity, funding stability, strategic planning, external environment, partnerships, communication, program adaptation, and program evaluation. These domains have been proven to affect the capacity for sustainability among public health programs (5); however, understanding of how these domains interact to improve program sustainability or how to determine how success in one domain might improve other domains is not yet understood.

This paper outlines the development and assessment of the *Program Sustainability Action Planning Model and Training Curricula*, an intervention which aimed to build capacity for sustainability in state tobacco control programs (TCPs) (16). Specifically, we developed the *Program Sustainability Action Planning Model and Training Curricula* as an action-oriented training model (defined by Kolb's experiential learning theory) (17) that addressed the internal and external program-related domains outlined in the Program Sustainability Framework (5) proven to affect the capacity for sustainability among public health programs. For the purposes of this paper, the “intervention” is the *Program Sustainability Action Planning Model and Training Curricula*. We also provide an assessment of the implementation strategy (i.e., use of experiential learning) of this intervention. Using results from this study, including participant perspectives regarding the implementation strategy including intervention component utility and suitability as well as programmatic outcomes, we propose an empirically-grounded conceptual model for implementing sustainability capacity-building interventions in public health settings. The proposed model builds upon and refines the original intervention model used in the development of the intervention, further specifying the implementation strategy, strategy-mechanism linkages, and effect modifiers (preconditions, mediators, and moderators).

## Methods

### Study design

The Plans, Actions, and Capacity to Sustain Tobacco Control (PACT) study utilized a multiphase approach to develop and assess the effectiveness of a novel intervention, the *Program Sustainability Action Planning Model and Training Curricula*, to increase the capacity for sustainability among state level tobacco control programs. In the first phase of the PACT study, the intervention was developed through a rigorous multidisciplinary literature review process and a series of expert consultations. We used SCOPUS, ERIC (ProQuest), PubMed, Education Full Text, and PsychINF databases to conduct a formative literature review to inform the development and evaluation of the training intervention. Specifically, we performed formative literature reviews regarding experiential models of learning (i.e., duration and components) and technical assistance (type and duration) to design the intervention. To design the evaluation of the intervention, we conducted formative review to assess previous metrics of experiential learning and technical assistance effectiveness. We also consulted with our PACT advisory team which was comprised of two academic experts in sustainability, two state tobacco control program directors, and three officials from the Centers for Disease Control and Prevention Office of Smoking or Health to determine the final *Program Sustainability Action Planning Model and Training Curricula*.

In the second phase of this study, a multiyear randomized control trial was conducted to assess the effectiveness in improving the capacity for sustainability [as defined by organization outcomes and Program Sustainability Assessment Test (PSAT) scores] among 24 State Level Tobacco Control Programs (TCP). Ultimately, 11 intervention and 12 control TCPs participated. The *Program Sustainability Action Planning Model and Training Curricula* was delivered to 11 TCPs through tailored workshops at baseline and ongoing, robust technical assistance through their participation in the multiyear randomized control trial (2018–2022). This paper presents only the results of the evaluation of the training and technical assistance delivered to the 11 intervention states as these data were used in defining the proposed conceptual model.

#### The program sustainability action planning model and training curricula

The intervention consisted of a two- day workshop to design a program sustainability action plan, two years of tailored technical assistance for implementing the action plan and sustainability outcome assessment. Participants of the workshops actively engaged in developing state TCP-specific sustainability action plans. Each state action plan outlined one or two domain-focused objectives, matched with time-specific activities to be shared across stakeholders present. One person at each workshop claimed responsibility for overseeing the implementation process. Sustainability plans were designed to be implemented over the course of two years. All Program Sustainability Action Planning Training workshops followed the same structure, but were tailored to each state depending on the Program Sustainability Framework domain chosen for the action plan. The two-day workshop involved the TCP staff and as well as a number of stakeholders (i.e., advocates, coalition members, voluntary organizations, grantees, local level health department staff) actively participating to design a sustainability action plan and develop an implementation strategy. Inclusion of and participation by all stakeholders engaged was an important component of the sustainability action plan development process and ensuring all components of the state TCPs were considered. Table 1 outlines the intervention components of this study.

**Table 1:** Components of the sustainability action planning training intervention.

Intervention Components	Dose
Introduction to Sustainability Webinar	1 h, 1 time
Access to Program Sustainability Assessment Test Sustainability Report	1 h, 3 time points
State program-level PSAT results follow-up correspondence	2 h, 3 time points
In-person Program Sustainability Action Planning Training	48 h, in person, 1 time point
Technical Assistance	1 h, 9–12 time points (quarterly for 3 years)
Access to final Sustainability Action Planning Training and Curricula tools and resources	as needed

#### Assessing intervention components

##### Quantitative data methods

Following receipt of the workshop, TCP staff and stakeholders (*n* = 106) completed a 29-item survey evaluating intervention components for their utility and suitability. Survey items assessing the workshop, workbook, and the instructors utilized a Likert-type scale with anchors “strongly disagree” (1) and “strongly agree” (5). Survey items were grouped by component, forming evaluation areas: Workshop Evaluation, Workbook and Workshop Usefulness, Workbook Satisfaction, and Instructor Evaluation. Survey items were based on the theory of change that allows for study on how a change (intervention) has influenced the design, implementation, and institutionalization of a program and were designed specifically to assess outcomes related to Kolb experiential learning components (17). Descriptive statistics were calculated at the item- and component-level to analyze evaluation survey data.

##### Qualitative data methods

###### Workshop and workbook evaluation

Included in the evaluation survey of the intervention were two open-ended questions, (1) *What were the three most important things you learned at this training? Please explain* and (2) *What suggestions do you have for improving this workshop?* A total of 220 answers were recorded in response to most important things learned at the training. Responses such as, *None*, and *Not applicable*, were excluded from analysis (*n* = 13). The remaining 207 responses were reviewed and grouped into themes. A total of 100 answers were recorded in response to suggestions for improvement. Responses such as, *None*, and *Not applicable*, were excluded from analysis (*n* = 37). The remaining 63 responses were reviewed and grouped into themes.

###### Technical assistance (TA) calls

From December 2018 to January 2022, staff from the 11 intervention TCPs participated in TA calls to assess their progress on implementing their sustainability plans and to determine challenges and resource needs. Each state received an average of 2 calls/year during their two years of study participation (*n* = 46). These calls lasted about 30–45 min and were audio recorded.

Each TA call record was professionally transcribed using an online service (Rev.com). Transcripts were reviewed for accuracy and uploaded to NVivo 20 (released in March 2020) for coding. We used an inductive approach for thematic analysis. We developed a codebook based on the items addressed in the TA calls. The codes and sub-codes of the initial codebook were revised throughout the coding of transcripts. The final codebook consisted of four codes and sixteen sub-codes.

For the coding process, three research team members coded transcripts until they reached substantial inter-rater reliability (kappa = .72) (18). Differences between coders were discussed and addressed. Remaining transcripts were coded by a single research team member. This work focuses on the themes from two parent codes: (1) developing capacity for sustainability and (2) overall study feedback.

#### Diagram modelling

Quantitative and qualitative intervention component data were fit to the original intervention model through an Agile Science-informed causal pathway diagram modeling process to propose a generalizable, empirically-grounded conceptual model for implementing sustainability capacity-building interventions in public health settings. An Agile Science informed process was utilized due to the focus on intervention modularity and condition specification. As outlined by Lewis et al. (19), in implementation research this process entails specifying implementation strategies, strategy-mechanism linkages, effect modifiers, and distal and proximal outcomes (19). In the present study, the research team carried out the diagram modeling process over multiple working sessions. The research team presented results to the PACT advisory team, to ensure face and content validity.

## Results

### Assessing intervention components

#### Quantitative results

##### Workshop evaluation

The workshop evaluation area comprised nine survey items assessing logistics, utility, suitability, and outcomes. At the component level, the workshop was favorably assessed by intervention participants (*M* = 4.24, SD = 0.81). At the item level, participants indicated that the workshop augmented their understanding of the action planning process and their capacity to move these plans forward (*M* = 4.34, SD = 0.69; *M* = 4.37, SD = 0.65), and was overall beneficial for their program (*M* = 4.35, SD = 0.69). Participants indicated that they planned to translate workshop learning objectives into their tobacco control work (*M* = 4.47, SD = 0.59). See Table 2.

**Table 2:** Program sustainability action planning model and training curricula evaluation.

Training Component and Evaluation Indicator	Mean	Standard Deviation
**Workshop Evaluation**		
The quality of this workshop was excellent	4.39	0.66
The length of the workshop was just right	4.26	0.86
The objectives were clearly articulated	4.34	0.76
The objectives were achieved	4.33	0.67
The workshop was beneficial for my program	4.35	0.69
I understand the process of action planning completely	4.34	0.69
I feel capable of helping move the action plan forward	4.37	0.65
I plan to use what I learned from this workshop in my tobacco control work	4.47	0.59
I could successfully complete an action plan without this workshop	3.36	1.02
**Workshop and Workbook Usefulness**		
Defining the program	4.21	0.74
Understanding sustainability	4.3	0.61
Reflecting on results	4.26	0.7
Building an action plan	4.49	0.61
Continuing progress	4.14	0.69
Appendix resources	3.89	0.83
**Workbook Satisfaction**		
Design	4.48	0.65
Organization of content	4.50	0.59
Legibility and ease of use	4.55	0.63
Clarity of activity instructions	4.41	0.65
**Instructor**		
Demonstrated a thorough knowledge of the subject matter	4.68	0.56
Were well prepared for class	4.75	0.56
Presented material in a clear and organized manner	4.67	0.53
Used effective teaching/facilitating techniques	4.58	0.66
Respected and encouraged other's viewpoints	4.86	0.35
Discussed how the information can be applied in an actual situation	4.57	0.62
Made time for questions, answers, and discussion	4.79	0.43

Survey items were measured using a Likert-type scale with anchors “strongly disagree” (1) and “strongly agree” (5). Data were collected from September 18 to October 19 from 106 recipients.

##### Workbook and workshop usefulness

*The workbook and workshop usefulness* area comprised 6 survey items assessing the usefulness of intervention component modules: Defining Program (*M* = 4.21, SD = 0.74), Understanding Sustainability (*M* = 4.30, SD = 0.61), Reflecting on Results (*M* = 4.26, SD = 0.70), Building an Action Plan (*M* = 4.49, SD = 0.61), Continuing Progress (*M* = 4.14, SD = 0.69), and Appendix Resources (*M* = 3.89, SD = 0.83). At the component level, participant responses indicate that the workbook and workshop were useful (*M* = 4.22, SD = 0.72) (see Table 2).

##### Workbook satisfaction

*The workbook satisfaction a*rea comprised 4 survey items assessing design (*M* = 4.48, SD = 0.65), content organization (*M* = 4.50, SD = 0.59), ease of use (*M* = 4.55, SD = 0.63), and instructional clarity (*M* = 4.41, SD = 0.65). At the component level, participants were highly satisfied with the workbook (*M* = 4.48, SD = 0.63) (see Table 2).

##### Instructor evaluation

To evaluate the workshop training instructor, we used seven survey items assessing their subject matter expertise and professionalism. At the item level, participants reported that workshop instructors demonstrated a thorough knowledge of the subject matter (*M* = 4.68, SD = 0.56), used effective teaching and facilitating techniques (*M* = 4.58, SD = 0.66), and discussed how workshop learning objectives could be applied to their tobacco control work (*M* = 4.57, SD = 0.62). At the component level, instructors were favorably assessed (*M* = 4.70, SD = 0.53) (see Table 2).

#### Thematic analysis results

##### Workshop and workbook evaluation

Overall, participants found that the sustainability training intervention provided them with a better understanding of program sustainability and improved their ability to develop sustainability action plans. Participants noted it was useful to have a common language around sustainability and a shared action plan to be completed collaboratively by the TCP and its partners.

*It was very important and helpful to have common language and definitions for the domains –State Health Department staff*

Participants also shared that the workshop enhanced their ability to assess the strengths and weaknesses of their program. The workshop also helped participants gain a better understanding of their partners, noting how valuable it was to have dedicated time with their partners and include them in their sustainability action plan.

*…just working through the actual action plan because we're all so busy and so being able to do that and get that started in that meeting. I don't how long it would have taken us to get that done otherwise. –State Health Department, Director*

Program managers emphasized the importance of the workshop in providing direction for their sustainability work throughout the study.

##### Technical assistance

The TCP managers often commented on the importance of robust, ongoing technical assistance to continue implementing their sustainability plan. Ongoing access to program sustainability resources and intervention-facilitated peer learning were frequently requested during TA calls to further implementation of their action plans.

*I think it's always been nice to have timely reminders to focus on the strategic planning process and make sure that we're continually looking at how we're tracking against those milestones, the way outlined in the sustainability plan. That's been really useful for me *…* –State Health Department, Program Manager*

Managers also felt that the alignment of their action plan with other grant requirements facilitated implementation. Strong partner engagement was also identified by program managers as a factor which augmented sustainability training and technical assistance effectiveness.

*I appreciate the technical assistance and all of the tools that you put together for us to use. It was really, really helpful and it helped catalyze some really robust conversations at some of the stakeholder meetings we've had *…  *–State Health Department, Program Manager*

Program managers identified several factors that interfered with their progress in implementing their plans throughout the study. Most notable were high levels of staff turnover, competing priorities, partnership challenges, and the COVID-19 pandemic. In discussing staff turnover, program managers shared they were responsible for covering the duties of the vacant positions in their TCP making it difficult to prioritize their action plan. They also noted the vacancies they experienced in their program were at times the individuals directly responsible for aspects of the action plan, which slowed down and at times halted their progress.

*While that position is vacant, I'm kind of doing double duty, which just makes it hard to be focused on longterm just because of dealing with the day to day. We still try to do that as much as we possibly can, but I'm definitely looking forward to getting that position filled so we can share that workload a little bit. –State Health Department, Policy Section Manager*

Regarding competing priorities, managers discussed the prioritization of funding and contractual agreements, as well as emergency bans on tobacco products which required immediate attention.

*Plus we had that emergency ban on vapor products, flavored vapor products that kind of inundated our time. So, we had to push a lot of our dates back, and so that's what we did. So, we spent a lot of time revising the action plan in terms of dates and really moving towards completing some of the activities that we had said that we would do by January. –State Health Department, Unit Manager*

Some program managers noted they struggled to maintain engagement with their partners throughout their participation in the study, leading to slower responses to communication and lack of follow through on action plan responsibilities.

*Trying to make sure that partners were continually engaged and that when they left the meeting, that they felt empowered enough to actually follow through on activities that have been discussed. Because sometimes we would have meetings and everybody would leave with what they were supposed to do but when we met again there hadn't been any movement on anything. –State Health Department, Program Director*

Finally, the COVID-19 pandemic impacted the overall capacity of TCPs, as public health staff were reassigned to work on pandemic response activities.

*And we also were impacted with COVID and capacity, so we definitely had a plan and we had a product to share with legislators, but one of our staff has been and remains full-time on COVID *…* –State Health Department, Program Manager*

*…I'm sure you've heard this from other people, but partners are dealing with COVID too… they don't have the time or ability to sit in on some of the meetings that they normally would with us. So that's been somewhat of an issue as well. –State Health Department, Program Director*

When asked about recommendations for sustainability training and technical assistance intervention improvement, participants wanted it to be more adaptable to the changing needs and priorities of their TCP. They also requested additional opportunities to interact with other states enrolled in the study to further peer learning.

*I think, as we go through the strategic planning process, if you have seen strategic plans from other States that you think look amazing, feel free to send them… if you've been working with other States and you're really impressed with the caliber of work that they're doing, they have innovative ideas, I definitely think it's a good idea to learn from the best so that we might be able to emulate. –State Health Department, Program Manager*

### The conceptual model

**Figure 1:**
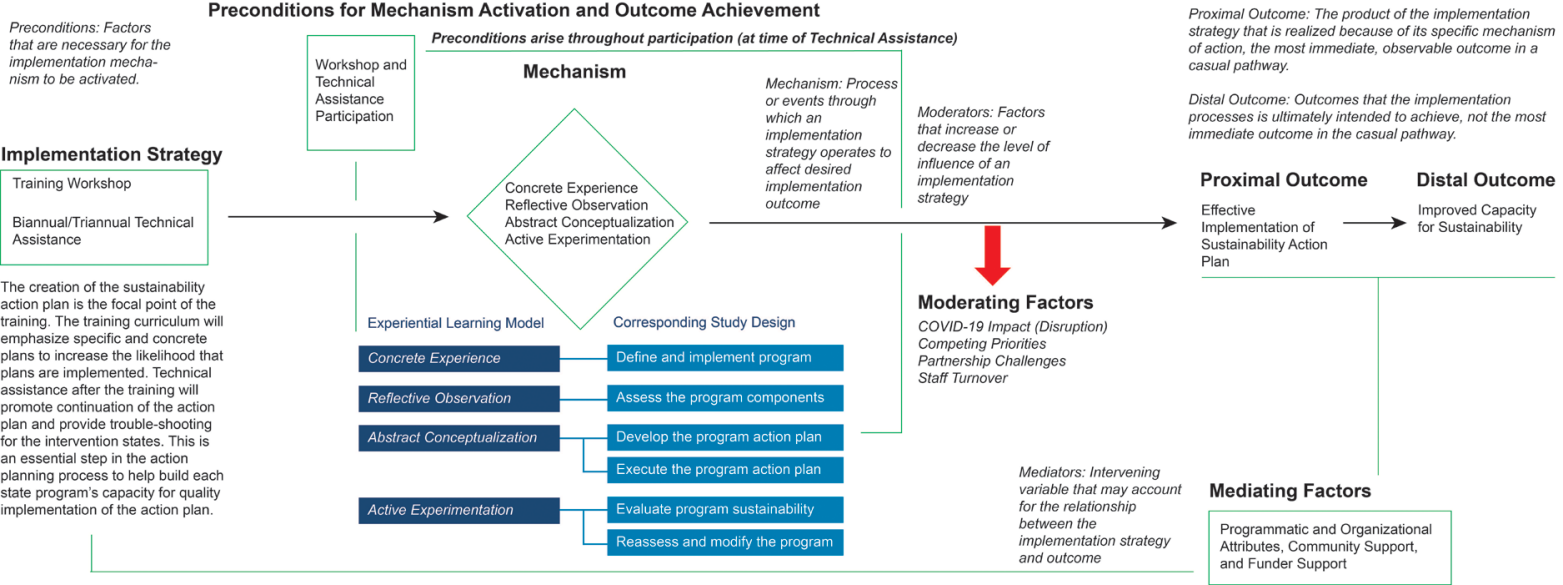
Conceptual model for building program sustainability in public health settings.

Using quantitative and qualitative data we engaged in an Agile Science-informed causal pathway diagram modeling process (19) to develop our conceptual model (Figure 1). The implementation strategy consisted of the *Program Sustainability Action Planning Model and Training Curricula*. The mechanism through which the Program Sustainability Action Planning Model and Training Curricula intervention was implemented was through action oriented, participatory training, as defined by Kolb's experiential learning theory (17). Both quantitative and qualitative results indicate the *Program Sustainability Action Planning Model and Training Curricula* was an effective intervention in developing and implementing a sustainability action plan.

Moderating factors or factors that increase or decrease the level of influence of the implementation of the state TCP sustainability action plans included high levels of staff turnover, competing priorities, partnership challenges, and the COVID-19 pandemic. While the pandemic was specific to this project's timing, other major public health events could be considered.

Mediating factors or variables that influence the outcome of the implementation strategy included programmatic and organizational factors, community partner support and funder support. Programmatic factors contributing toward the success of sustainability action plan implementation included dedicated time to work on sustainability plan activities and leadership support in continuing to work on the plans in spite of other competing priorities. Those state TCPs experiencing staff vacancies, especially involving those staff responsible for implementing the plan, slowed the ability to complete plan activities. Qualitative results indicated TCPs with strong partner engagement throughout their implementation process were more successful in completing their objectives and goals outlined in their sustainability plans. Likewise, some programs were unable to complete certain objectives outlined because they struggled to maintain engagement of their partners throughout their participation in the study. Competing priorities or lack of budgetary funding support for TCPs deterred progress on TCP sustainability action plan implementation (see Figure 1).

## Discussion

The present study makes several important contributions to implementation science. Notably, the present study documents the development and implementation of the novel *Program Sustainability Action Planning Model and Training Curricula*, one of the first proposed training interventions for improving the capacity for program sustainability in public health. We also propose an empirically-grounded conceptual model for implementing sustainability capacity-building interventions in public health settings. The proposed model builds upon previous work and specifies the implementation strategy, strategy-mechanism linkages, and effect modifiers (preconditions, mediators, and moderators).

Our results indicate that *Program Sustainability Action Planning Model and Training Curricula* was an effective capacity building intervention in developing and implementing a sustainability action plan. Those in receipt of the *Program Sustainability Action Planning Model and Training Curricula* assess it favorably in regards to its utility and suitability, across all evaluation areas. Thematic analysis further qualified quantitative results: participants indicated that the *Program Sustainability Action Planning Model and Training Curricula* enhanced their understanding of program sustainability and related program-specific characteristics as well as the role of TCP partners. In addition, throughout the study, program managers emphasized the importance of the workshop in providing direction for their sustainability work and the value of robust, ongoing technical assistance. Ongoing access to program sustainability resources and intervention-facilitated peer learning and partner engagement were also noted as factors augmenting *Program Sustainability Action Planning Model and Training Curricula* intervention component effectiveness.

We used both qualitative and quantitative results in refining a conceptual model for implementing sustainability capacity-building interventions in public health settings. Other studies focused on understanding sustainability have recognized similar components (mediators and moderators) identified in our proposed model but none have considered the complete process including the implementation strategy and mechanism for which to plan for and improve program sustainability. For instance, when utilizing the Normalization Process Theory in evaluating the implementation of an evidence-based violence screening model, Hooker et al., found several organizational components that mediated program “normalization” or programmatic sustainability including lack of staff capacity (15). Similar to our results, authors also noted the importance of partner and community interaction (collective action) in achieving desired results. Finally, authors also noted the importance of tracking (reflective monitoring) results. This is similar to the idea of the inclusion of active tracking of sustainability action plans in this study. Though not broadly applied, The Dynamic Sustainability Framework also defines similar components to our conceptual model. Specifically, the idea that systems are not static, but rather dynamic forcing programs to be adaptive and ready to respond to a constantly changing environment to be sustainable (13). In addition to the advent of new programmatic components or evidence invoking the need for change of a program, there exist external factors and events that require a dynamic response by programs. COVID-19 and changes in tobacco regulatory requirements were found to influence sustainability in this study.

### Limitations

There are several limitations to this study. First, although evidence-informed and developed through a systematic process, the proposed model has not been empirically tested. The present study also utilized programmatic data and the perspectives of individuals at 11 state TCPs. Although this is of sufficient size to statistically evaluate the intervention for its effectiveness, findings may not be completely generalizable across all state TCPs. Further work is therefore needed to establish generalizability. Finally, our data were generated throughout the COVID-19 pandemic, and therefore are not unaffected by the unprecedented conditions the event produced. Throughout this time state TCPs operated in nonroutine ways, and the extent to which emergent phenomena in our study were products of this is unknown. While we feel that any public health event might disrupt the state health department system, one may not have the same magnitude effect.

## Conclusion

By establishing a method for action planning and technical assistance for program sustainability, the present study supports public health programs broadly in their understanding of and achievement in sustainability, an outcome that has become increasingly critical given the environmental complexity. In addition, this work advances the field of study regarding action planning and technical assistance, which contributes to implementation science beyond the topic area of sustainability. Finally, the present study advances implementation science by establishing an empirically-grounded conceptual model for program sustainability and related capacity-building interventions. Considering the paucity of research in this understudied and undefined topic area, this is a significant contribution that can serve as a framework for similar intervention designs and implementation efforts. Future research in the application of this framework will be beneficial in defining its utility and refining its components.

## Data Availability

The raw data supporting the conclusions of this article will be made available by the authors, without undue reservation.
